# Supplementary material for: Mycobacterium leprae promotes triacylglycerol de novo synthesis through induction of GPAT3 expression in human premonocytic THP-1 cells
Source: PLoS One. 2021 Mar 26;16(3):e0249184. doi: 10.1371/journal.pone.0249184 (PMC7997041; doi:10.1371/journal.pone.0249184)
Supplement: S1 Table — (DOCX) [file pone.0249184.s001.docx]

**S1 Table. List of primers used in RT-PCR.**

| **Gene name** | **Forward (5’ - 3’)** | **Reverse (5’ - 3’)** | **Length (bp)** |
| --- | --- | --- | --- |
| *GPAT1* | AACCCCAGTATCCCGTCTTT | CAGTCACATTGGTGGCAAAC | 144 |
| *GPAT2* | GGCTGACGGAGGAGATACTG | AGTTGTGCCAGGTGTGTGAG | 179 |
| *GPAT3* | ACAGCAGCCTCAAAAACTGG | CAATGGGGGAAGTATGGTTG | 153 |
| *GPAT4* | TGCCAAATGGGAGGTTTAAG | GCCACCATTTCTTGGTCTGT | 128 |
| *hsp-70* | TACCGACATTTCCGCGATAAAGTCGGCA | CGTCAACCACATCGTCAGTAGA | 157 |
| *ML0435* | GACCTCGTTCTTCTCTTCTTCG | TGATTCTTCGTGACAACCTCG | 156 |
| *ML1474* | AATGCTAGCATGGTGATGTCG | TATTGACGTTCGTCACTACGG | 137 |
| *ML1475* | GCAACCATCTGATACATGCG | CATGCTGGACATTGATCACC | 142 |
| *ML1476* | GCAACCATCTGATACATGCG | CATGCTGGACATTGATCACC | 198 |
| *ML1636* | CAACCGAGTTACTGGATGTGG | GGTTCAGTGGTGTTGTTAGCC | 205 |
| *ML1752* | GGTCTGTTGCAGGTGATTCC | ATATCACCACCGAGGTTCTCG | 152 |
| *ML2492* | CCTCGATACGTGAGTTGTTCC | TGGTCTGATGGTATCGTCACC | 126 |
| *ACTB* | AGCCATGTACGTAGCCATCC | TGTGGTGGTGAAGCTGTAGC | 220 |
