# Supplementary figures and images for: Mycobacterium leprae promotes triacylglycerol de novo synthesis through induction of GPAT3 expression in human premonocytic THP-1 cells
Source: PLoS One. 2021 Mar 26;16(3):e0249184. doi: 10.1371/journal.pone.0249184 (PMC7997041; doi:10.1371/journal.pone.0249184)

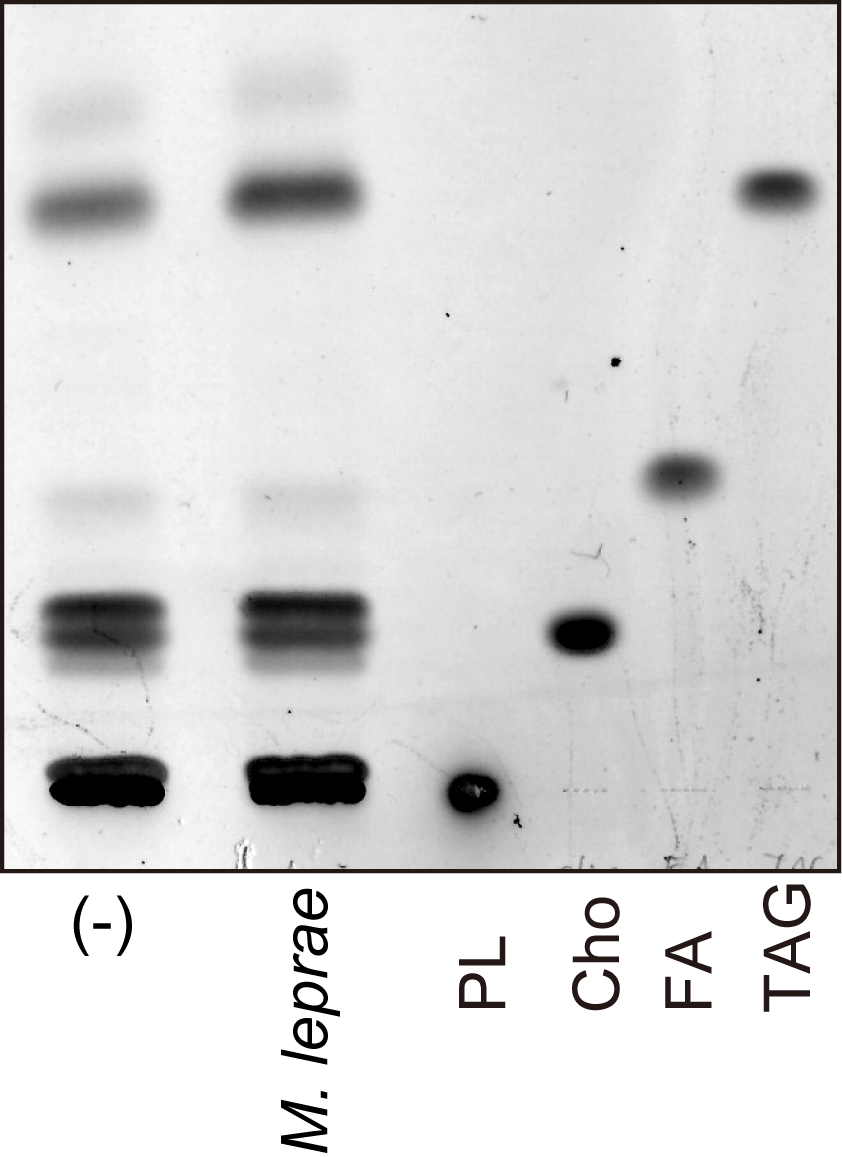

Supplement: S1 Fig — THP-1 cells (3 × 10^6^) were cultured in 6-well plates with live M. leprae (MOI: 20) for 24 h. Total lipids extracted from cells with 20 nmol of control lipids (PL, Cho, FA and TAG) were spotted on an HPTLC plate. After separation, the plate was stained with a charring solution containing 10% CuSO4 and 8% H3PO4 and heated at 180°C for 10 min. (TIF) [file pone.0249184.s002.tif]

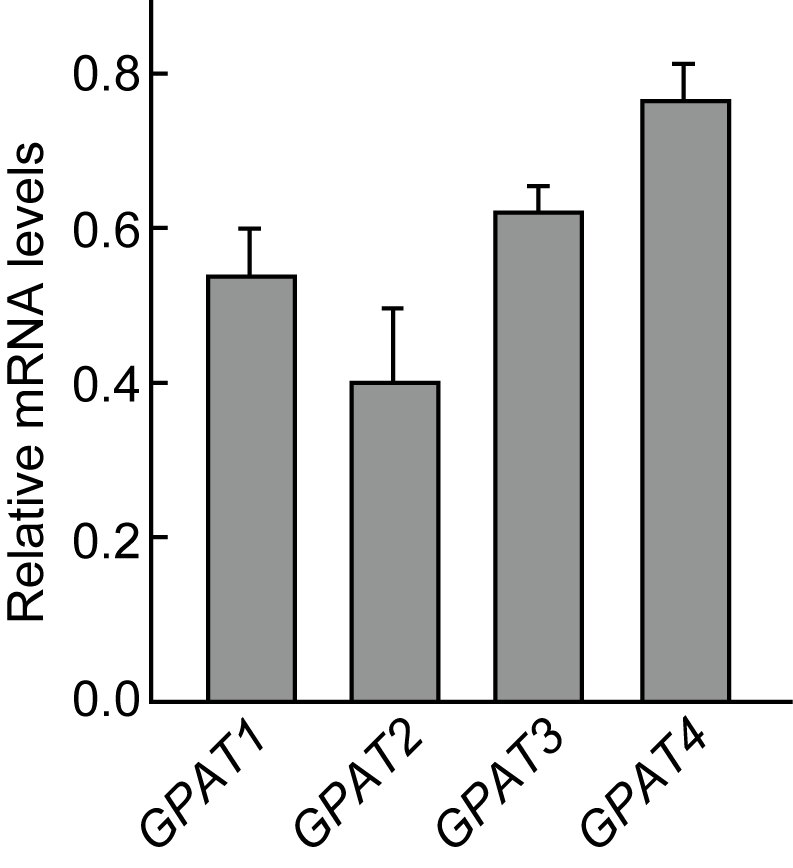

Supplement: S2 Fig — Total RNA was extracted and the expression level of GPAT isoforms was evaluated by qRT-PCR. The results were normalized relative to ACTB levels. Each bar represents the mean ± S.D in triplicate. (TIF) [file pone.0249184.s003.tif]

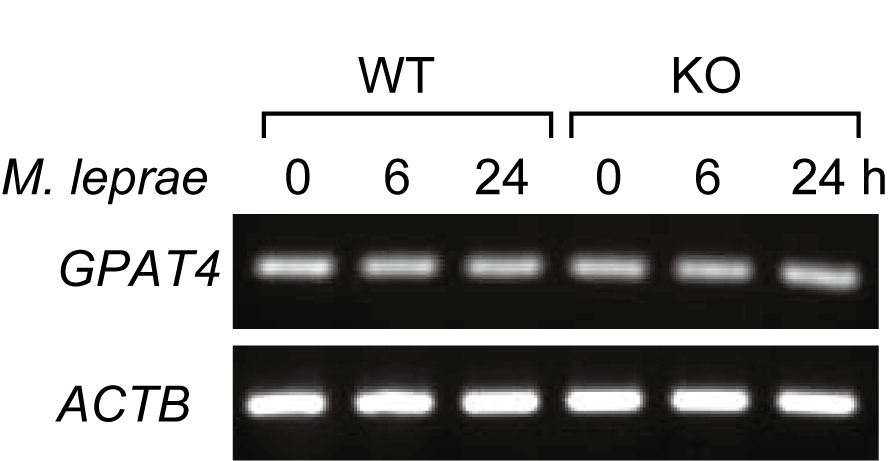

Supplement: S3 Fig — WT and GPAT3 KO cells (3 × 10^6^) were cultured in 6-well plate and infected with live M. leprae (MOI: 50). After incubating for the indicated time, total RNA was purified and RT-PCR analysis was performed. (TIF) [file pone.0249184.s004.tif]

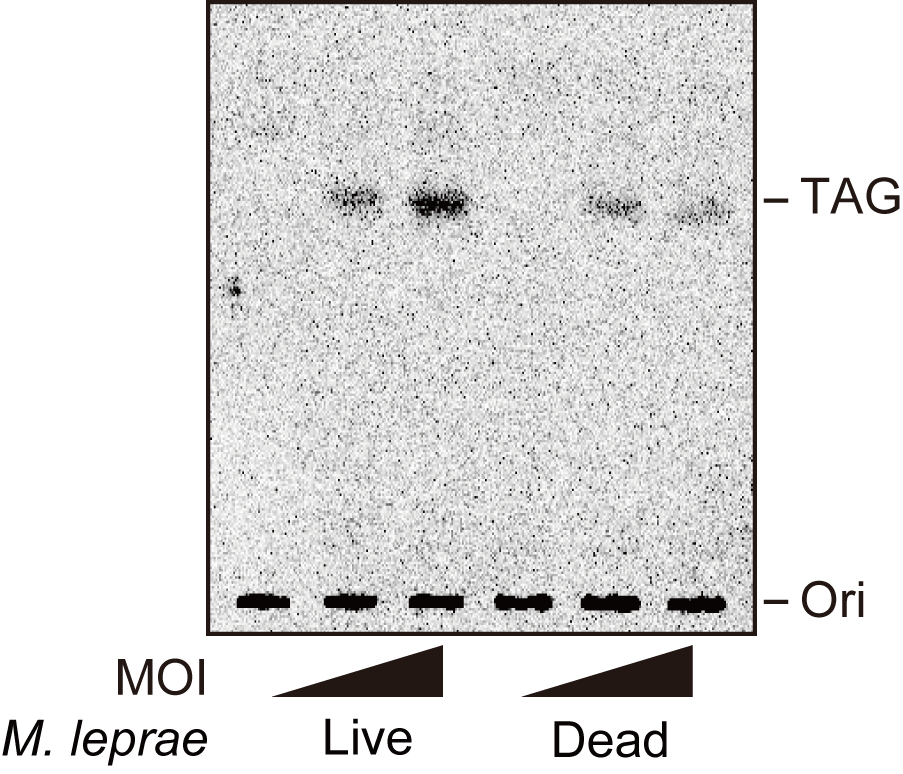

Supplement: S4 Fig — Wild-type THP-1 cells were inoculated with either live or heat-killed M. leprae (MOI: 10 and 50), then cultured with 0.2 μCi of [14C] stearic acid for 16 h. M. leprae was isolated and extracted lipids were separated by TLC to evaluate radioactivity. (TIF) [file pone.0249184.s005.tif]

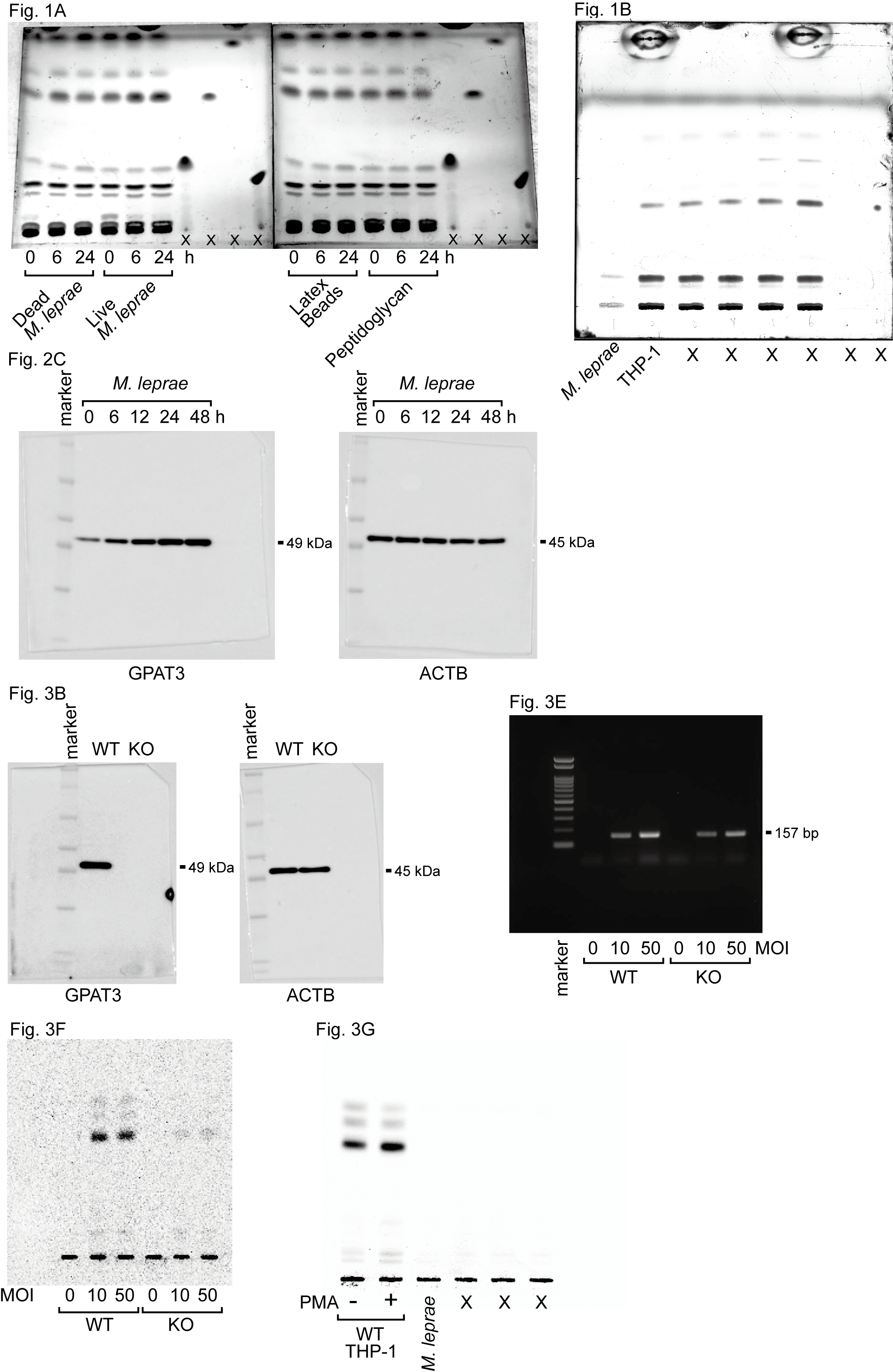

Supplement: S1 Raw images — (TIF) [file pone.0249184.s006.tif]

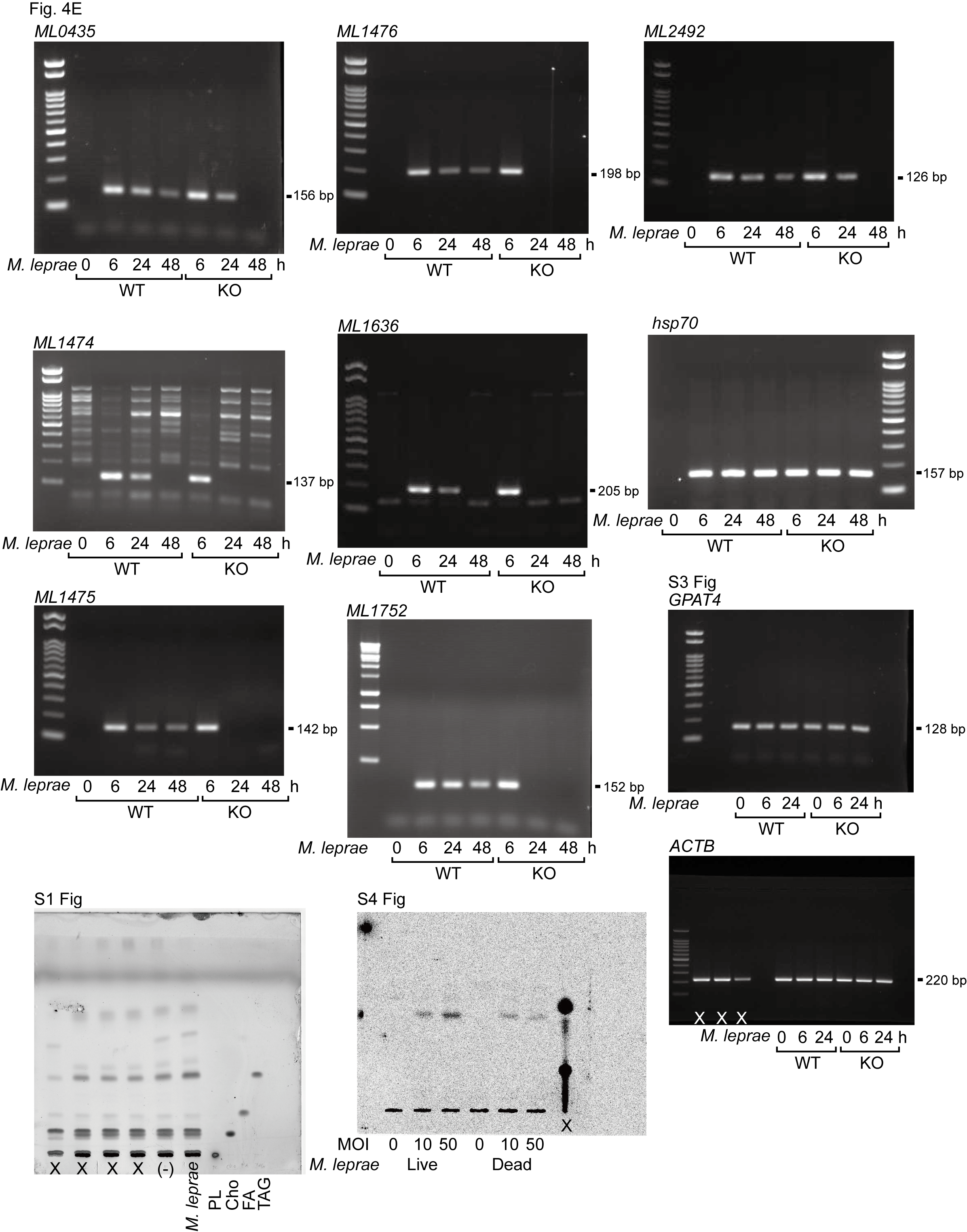

Supplement: S2 Raw images — (TIF) [file pone.0249184.s007.tif]
