# Supplementary material for: Mycobacterium leprae promotes triacylglycerol de novo synthesis through induction of GPAT3 expression in human premonocytic THP-1 cells
Source: PLoS One. 2021 Mar 26;16(3):e0249184. doi: 10.1371/journal.pone.0249184 (PMC7997041; doi:10.1371/journal.pone.0249184)
Supplement: Attachment — Submitted filename: Re-response to reviewers+.docx [file pone.0249184.s009.docx]

**Reviewer 2**

**The authors have responded to most comments and made the appropriate changes in the manuscript. However, few additional changes need to be included in the final version, as follows:**

Response:

We thank the reviewer for the comments. We have addressed the reviewer’s concerns at below.

**1. Authors should include details of the assay performed with dead bacteria to test the potential unspecific adsorption of TAG to *M. leprae* in the Materials and Methods section, such as the number of cells and bacteria used.**

Response:

We have included the experiment performed with wild-type THP-1 cells infected with dead *M. lepra* in S4 Fig, as well as the experiment using live *M. leprae* incubated with PMA-treated THP-1 cell lysate in Fig. 3G. Detailed protocol was described in the Material and Methods and in Figure legends accordingly as follows.

Materials and Methods, page 9, lines 189-191:

“*THP-1 cells (1 × 10^6^) were inoculated with ether live or heat-killed M. leprae (MOI: 10 and 50) for 24 h, then incubated with 0.2 μCi [^14^C] stearic acid (American Radiolabeled Chemicals, Saint Louis, MO) for 16 h at 37°C with 5% CO_2_.*”

Fig. 3 legend, pages 15-16, lines 343-345:

“*(E and F) WT and GPAT3 KO THP-1 cells were cultured in medium containing 0.2 μCi of [^14^C] stearic acid for 16 h after M. leprae infection (MOI: 10 and 50).*”

S4 Fig legend, page 30, lines 664-666:

“*Wild-type THP-1 cells were inoculated with either live or heat-killed M. leprae (MOI: 10 and 50), then cultured with 0.2 μCi of [^14^C] stearic acid for 16 h. M. leprae was isolated and extracted lipids were separated by TLC to evaluate radioactivity.*”

**2. In figure 3C, images of uninfected wild type and KO cells stained with lipidTOX should be included.**

Response:

According to the suggestion, we added images of the LipidTOX fluorescent staining of uninfected wild type and KO cells in Fig. 3C. The Figure legend was modified accordingly.

**3. The sentence “In this study, we performed a comprehensive lipid analysis using HPTLC to show that TAG is the main lipid that accumulates in lipid droplets in *M. leprae*-infected macrophages” needs to be changed. Their study did not perform “a comprehensive lipid analysis “. Moreover, as pointed out in the first round of review, macrophages should be replaced by THP-1 cells.**

Response:

According to the Reviewer’s suggestion, we have rephrased a sentence in the Discussion section as follows (page 19, lines 407-409):

“*In this study, we performed a qualitative lipid analysis using HPTLC to show that TAG is the main lipid that accumulates in lipid droplets in M. leprae-infected THP-1 cells.*”

**4. In the sentence “Primary cells infected with *M. leprae* are known to be rich in cholesterol and cholesterol esters, but not THP-1 cells [27]”, reference 27 refers just to the first part, not to THP-1 cells. Please make changes accordingly.**

Response:

We have corrected the position of the reference as follows (pages 20-21, lines 452-456).

“*Additionally, the cells used in the previous report were primary cultures of human macrophages, and primary cells infected with M. leprae are known to be rich in cholesterol and cholesterol esters [**27**]. Differences in the lipid moieties accumulated in response to M. leprae infection between primary cells and THP-1 cells need to be clarified in the future.*”

**5. In the section where they speculate about the possible involvement of PPARγ, they should mention the study by Diaz Acosta CC et al. 2018, in which they show that this transcriptional fact** **or is induced by M. leprae in infected Schwann cells and participates in host cell lipid accumulation.**

Response:

We thank the reviewer for pointing out this issue. We have cited the suggested paper (Diaz Acosta CC *et al*., 2018) as well as ours (Luo, *et al*., 2020) describing the involvement of PPARγ in *M. leprae* infection in the Discussion section as follows (page 21, lines 466-471):

“*Recently, we have reported that activation of PPARγ and PPARδ is important for lipid accumulation in M. leprae-infected THP-1 cells [Luo, 2020 #62]. In Schwann cells, phenolic glicolipid-1 (PGL-1) of M. leprae promoted lipid droplet formation by activating crosstalk between CD206 and PPARγ [Diaz Acosta, 2018 #32]. Therefore, M. leprae might utilize the signal transduction pathway(s) mediated by PPARγ to induce GPAT3 expression in infected cells*.”
